# The Spatial Distribution of Plaque Vulnerabilities in Patients with Acute Myocardial Infarction

**DOI:** 10.1371/journal.pone.0152825

**Published:** 2016-03-31

**Authors:** Guian Zheng, Yuxin Li, Tadateru Takayama, Toshihiko Nishida, Mitsumasa Sudo, Hironori Haruta, Daisuke Fukamachi, Kimie Okubo, Yoshiharu Higuchi, Takafumi Hiro, Satoshi Saito, Atsushi Hirayama

**Affiliations:** 1 Department of Advanced Cardiovascular Imaging, Nihon University School of Medicine, Tokyo, 173-8610, Japan; 2 Department of Cardiology, Zhangzhou Hospital Affiliated to Fujian Medical University, Zhangzhou, 363000, Fujian, China; 3 Division of Cardiology, Department of Medicine, Nihon University School of Medicine, Tokyo, 173-8610, Japan; Shenzhen institutes of advanced technology, CHINA

## Abstract

**Objective:**

Although the plaque characteristics have been recognized in patients with acute myocardial infarction (AMI), the plaque spatial distribution is not well clarified. Using color-mapping intravascular ultrasound (iMAP-IVUS), we examined culprit lesions to clarify plaque morphology, composition and spatial distribution of the sites of potential vulnerability.

**Methods:**

Sixty-eight culprit lesions in 64 consecutive AMI patients who underwent angiography and IVUS examinations before intervention were analyzed. Plaque morphology and composition were quantified with iMAP-IVUS. The spatial distribution of the sites of potential vulnerability was assessed with longitudinal reconstruction of the consecutive IVUS images. The plaque characteristics were also compared between ruptured and non-ruptured lesions, and between totally occlusive (TO) and non-TO lesions.

**Results:**

The sites with maximum necrotic area (maxNA), maximum plaque burden (maxPB) and most severely narrowed (minimal luminal area, MLA) were recognized vulnerability. In the majority of cases, maxNA sites were proximal to the maxPB sites, and MLA sites were distal to the maxNA and maxPB sites. Ruptures usually occurred close to maxNA sites and proximal to maxPB and MLA sites. The average distance from the site of rupture to the maxNA site was 0.33 ± 4.04 mm. Ruptured lesions showed significant vessel remodeling, greater plaque volume, and greater lipidic volume compared to those of non-ruptured lesions. Both the length and plaque burden (PB) of TO lesions were greater than those of non-TO lesions.

**Conclusions:**

Instead of overlapping on maxPB sites, most maxNA sites are proximal to the maxPB sites and are the sites most likely to rupture. Plaque morphology and composition play critical roles in plaque rupture and coronary occlusion.

## Introduction

Acute myocardial infarction (AMI) is commonly caused by atherosclerotic plaque rupture or endothelial erosion with superimposed thrombosis, either of which results in abrupt coronary artery occlusion [1,2]. Although plaque ruptures have often been located in the proximal segments of the coronary arteries [3–6], the reasons for rupture at these locations are not clear. Intravascular ultrasound (IVUS) studies have shown that lesions with an extensive plaque burden (PB) and causing compensatory positive remodeling are vulnerable and prone to rupture [7–11]. However, the characteristics of ruptured plaque have not been fully described.

There is increasing evidence that AMI patients with totally occlusive (TO) culprit arterial lesions have worse short- and long-term clinical outcomes, such as reinfarction and death from coronary disease, than those of patients with non-totally occlusive (non-TO) lesions [12–17]. However, differences in plaque features between TO lesions and non-TO lesions remain to be elucidated.

iMAP (Boston Scientific, Marlborough, MA) is a newly developed color-mapping IVUS tissue characterization system based on pattern recognition of the radio frequency (RF) signals and can provide quantitative analysis of plaque composition [18–20]. We conducted a retrospective volumetric analysis of iMAP-IVUS images to investigate the spatial distribution of the sites of vulnerability and rupture. We also compared plaque morphology and composition between ruptured and non-ruptured lesions and between TO and non-TO lesions.

## Methods

### Ethics Statement

This retrospective study was approved by the Nihon University Itabashi Hospital Institutional Review Board (RK-150609-5) and conducted according to the principles of National Ethical Guidelines for Medical and Health Research Involving Human Subjects. All of the participants provided signed, informed consent at admission, before their data were stored in the hospital database.

### Study Patients

The study group comprised 64 consecutive AMI patients with 68 culprit lesions. These patients were identified from among 84 AMI patients admitted to our institution for emergency percutaneous coronary intervention (PCI) during the period February 2013 through April 2014. All had undergone IVUS examination before PCI. The diagnosis of AMI, which included ST-segment elevation myocardial infarction (STEMI) or non-STEMI, was based on significant elevation of at least 1 biomarker of myocardial necrosis (troponin I/T or creatine kinase-MB) in combination with a history of prolonged acute chest pain and characteristic electrocardiogram changes. Culprit coronary lesions were identified on the basis of angiographic lesion morphology, electrocardiographic findings and abnormal left ventricular wall motion (shown by echocardiography or left ventriculography). Patients not included in the study were those for whom manual pullback of the IVUS catheter was necessary, the IVUS images were of poor quality, stent restenosis occurred or vein graft lesioning or extensive coronary artery calcification was found.

### Angiographic Analysis

Coronary angiography was performed via the radial or femoral approach after intracoronary administration of nitroglycerin. Stenosis was quantified as follows: (reference vessel diameter−minimum lumen diameter) / (reference vessel diameter) ×100 (%). Lesions causing 100% stenosis were classified as TO lesions, and lesions causing < 100% stenosis were classified as non-TO lesions.

### Acquisition and Analysis of IVUS Images

After intracoronary administration of nitroglycerin, a mechanical rotating 40-MHz IVUS transducer (Atlantis^™^ SR Pro, Boston Scientific) was advanced beyond the culprit lesion after guidewire passage or after dilatation with a 1.5–2.0 mm diameter balloon and then pulled back to the aorto-ostial junction with an automatic pullback device at a rate of 0.5 mm/s. The IVUS images with the RF signals were stored on DVD for offline analysis.

Quantitative analysis of the IVUS images was performed according to criteria set forth in the American College of Cardiology Clinical Expert Consensus Document on IVUS [21]. All measurements were performed independently by an experienced analyst who was blinded to patients’ clinical characteristics and angiographic findings. The borders of the external elastic membrane (EEM) and lumen at the sites of target lesions were traced manually by means of IVUS analysis software (echoPlaque 3.0, INDEC Systems, Santa Clara, CA, USA). The EEM area was considered the vessel area. The plaque area was calculated as the EEM area minus the lumen area, and PB was calculated as the plaque area divided by the EEM area. A plaque ≥ 0.5 mm in thickness was regarded as a lesion. Lesions separated by ≥ 2 mm of normal vessel were considered discrete lesions. Lesion length was taken as the distance between the lesion’s proximal and distal edges. The minimal luminal area (MLA) was defined as the smallest luminal area inside the lesion. A plaque rupture was defined as a cavity that was in contact with the lumen with an overlying residual fibrous cap fragment [22]. In iMAP-IVUS analysis of plaque composition, various aspects of the RF signal are processed by autoregressive modeling and matched to a database of known RF signal profiles containing the characteristics of 4 basic tissue types [23]. The tissue components of the plaques were classified into the 4 basic types as fibrotic (green), lipidic (yellow), necrotic (red), and calcified (blue). After tracing the EEM and lumen of all involved iMAP-IVUS images from the distal to the proximal direction, the software automatically calculated lesion length, lumen area and volume, vessel area and volume, plaque area and volume, PB, fibrotic plaque area and volume, lipidic plaque area and volume, necrotic plaque area and volume and calcified plaque area and volume. To compensate for the effect of various lesion lengths on the volumetric variables, length-adjusted volumetric variables were used in the study. For example, length-adjusted lumen volume was calculated as: lumen volume divided by lesion length and then multiplied the median length of all 68 culprit lesions [24].

### Statistical Analyses

Categorical variables are shown as numbers or frequencies, and continuous variables are shown as mean ± SD. Comparisons were made between TO lesions and non-TO lesions and also between ruptured plaques and non-ruptured plaques. Between-group differences in categorical variables were analyzed by chi-square test or Fisher's exact test. Continuous variables were tested for normality of distribution by Kolmogorov-Smirnov test. Between-group differences in normally distributed variables were analyzed by unpaired Student’s t-test and in non-normally distributed variables were analyzed by Mann-Whitney U test or Kruskal-Wallis test. Association between various IVUS variables was assessed by simple linear regression analysis. All statistical analyses were performed with JMP 10.0 software (SAS institute, Cary, NC). A p value < 0.05 was considered statistically significant.

## Results

### Patient Characteristics

Patients’ clinical characteristics are shown in Table 1. Sixty-nine percent of the patients were male, and 86% had a STEMI. Prior myocardial infarction and a prior PCI were significantly more prevalent among patients with ruptured plaques than among those with non-ruptured plaques. The prevalence of dyslipidemia differed, though not significantly, between the TO group patients and non-TO group patients (46% versus 22%, *p* = 0.05), but there was no difference in age, sex or other coronary risk factors. STEMI was more prevalent in the TO group than in the non-TO group (95% versus 70%, respectively; *p* = 0.005).

**Table 1:** Clinical characteristics of the total patients, patients with ruptured versus non-ruptured plaques, and patients with TO versus non-TO lesions.

	All patients	Rupture	Non-rupture		TO	Non-TO	
	(n = 64)	(n = 26)	(n = 38)	*p* Value*	(n = 41)	(n = 23)	*p* Value**
Age (years)	66.1 ± 13.5	68.4 ± 2.6	64.5 ± 2.2	0.26	64.3 ± 14.9	69.2 ± 10.2	0.17
Male sex	44 (69)	17 (65)	27 (71)	0.63	25 (61)	19 (83)	0.07
BMI (kg/m^2^)	24.1 ± 3.8	23.3 ± 0.7	24.6 ± 0.6	0.18	24.3 ± 4.0	23.8 ± 3.6	0.57
Current Smoking	38 (59)	15 (58)	23 (61)	0.82	23 (56)	15 (65)	0.48
Hypertension	42 (66)	18 (69)	24 (63)	0.62	24 (59)	18 (78)	0.11
Diabetes	29 (45)	12 (46)	17 (45)	0.91	16 (39)	13 (57)	0.18
Dyslipidemia	24 (37)	10 (38)	14 (37)	0.90	19 (46)	5 (22)	0.05
Family history of CAD	9 (14)	5 (19)	4 (11)	0.33	6 (15)	3 (13)	0.86
Prior myocardial infarction	5 (8)	5 (19)	0	**0.005**	3 (7)	2 (9)	0.84
Prior PCIs	5 (8)	5 (19)	0	**0.005**	3 (7)	2 (9)	0.84
Plaque rupture	26(41)	26	0	NA	17 (42)	9 (38)	0.69
Clinical presentation							
STEMI	55 (86)	23 (88)	32 (84)		39 (95)	16 (70)	
Non-STEMI	9 (14)	3 (12)	6 (16)	0.63	2 (5)	7 (30)	**0.005**

Values are mean ± SD or n (%). CAD, coronary artery disease; PCI, percutaneous coronary intervention; STEMI, ST-segment elevation myocardial infarction. TO, totally occlusive; BMI, body mass index;

*Rupture versus non-rupture.

**TO versus non-TO.

### Angiographic Findings

Sixty of the 64 patients had 1 culprit lesion, and the remaining 4 (6%) patients had 2 culprit lesions. Of the 68 lesions, 42 (62%) were TO lesions and 26 (38%) were non-TO lesions. Angiographically determined distribution of the culprit lesions is shown in Table 2. Fifty percent of the lesions were located in left anterior descending artery. There was no significant difference in lesion location between the TO group and the non-TO group.

**Table 2:** Angiographically determined distribution of the culprit lesions.

Lesion location	All lesions	TO lesions	Non-TO lesions	*p* Value*
	(n = 68)	(n = 42)	(n = 26)	
LAD, n (%)	34 (50)	22 (52)	12 (46)	0.62
RCA, n (%)	23 (34)	15 (36)	8 (31)	0.67
LCX, n (%)	11 (16)	5 (12)	6 (23)	0.38

*Total occlusion versus non-total occlusion lesions.

LAD: left anterior descending artery; RCA: right coronary artery; LCX: left circumflex.

### Spatial Distribution of Areas of Vulnerability and Ruptures

**Fig 1:**
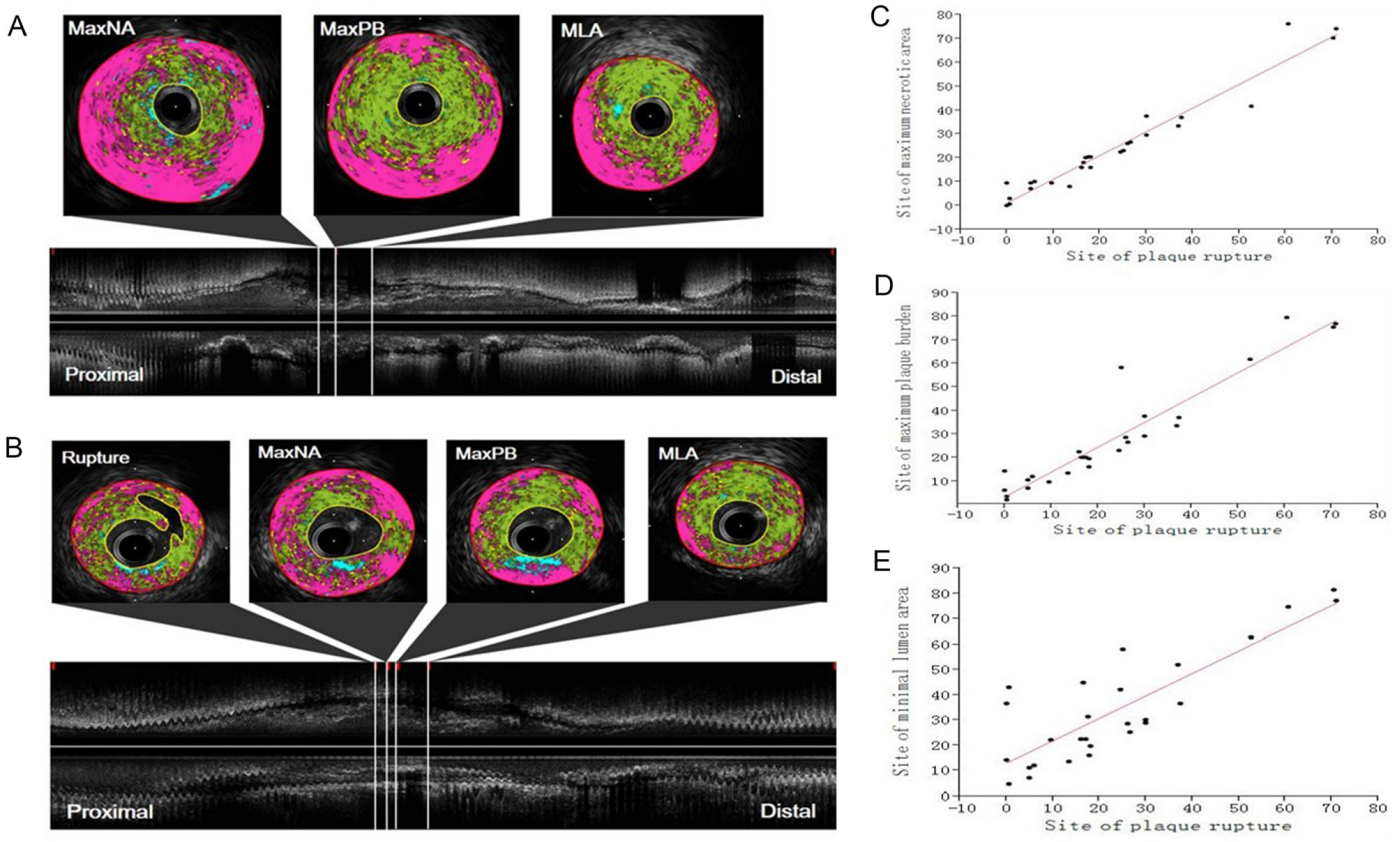
**A**: Representative iMAP color-mapping images of the maxNA site, the maxPB site and the MLA site, and longitudinal reconstruction of the consecutive IVUS images from the proximal edge to distal edge of culprit lesion. **B**: Representative iMAP color-mapping images of the site of rupture, the maxNA site, the maxPB site and the MLA site, and longitudinal reconstruction of the consecutive IVUS images from the proximal edge to distal edge of ruptured lesion. Scatter plots showing correlation between locations of plaque rupture and locations of the maximum necrotic area **(C)**; correlation between locations of plaque rupture and locations of maximum plaque burden **(D)**; and correlation between locations of plaque rupture and locations of minimum lumen area **(E)**. Locations are plotted as distances from the proximal edges of ruptured lesions. maxNA: maximum necrotic area; maxPB: maximum plaque burden; MLA: minimum lumen area; MD: mean distance.

Using longitudinal reconstruction of the consecutive IVUS images, we assessed the spatial relations between sites of potential vulnerability within the culprit lesions by measuring the distance between the sites of maximum necrotic area (maxNA), maximum PB (maxPB), MLA and rupture. Overall, the distance from the proximal edge of the lesion to the site of maxNA was 18.4 ± 17.5 mm, to the site of maxPB was 21.8 ± 17.7 mm and to the MLA site was 28.2 ± 20.1 mm. In the majority of cases, the most stenotic sites (MLAs) were distal to the maxNA and maxPB sites (Fig 1A). Specifically, 75% of maxNA sites were proximal to the MLA sites, and 79% of maxPB sites were proximal to (54%) or overlapped (25%) the MLA sites (Table 3). For the spatial relations between maxNA sites and maxPB sites, 72% of maxNA sites were proximal to (62%) or overlapped (10%) the maxPB sites (Table 4).

**Table 3:** Overall spatial relations between the maxNA and maxPB sites and the MLA site.

	maxNA site		maxPB site	
	n (%)	Distance (mm)	n (%)	Distance (mm)
Proximal to the MLA site	51 (75)	14.4 ± 14.3	37 (54)	12.6 ± 12.7
On the MLA site	1 (1)	0	17 (25)	0
Distal to the MLA site	16 (24)	4.1 ± 6.8	14 (21)	2.0 ± 2.5

Distances are mean ± SD for the total lesions. maxNA: maximum necrotic area; maxPB: maximum plaque burden; MLA: minimum lumen area.

**Table 4:** Distance from the maxNA site to the maxPB site in all lesions.

Location of the maxNA site	n (%)	Distance (mm)
Proximal to the maxPB site	42 (62)	7.8 ± 9.8
At the maxPB site	7 (10)	0
Distal to the maxPB site	19 (28)	5.00 ± 8.8

Distances are mean ± SD. maxNA, maximum necrotic area; maxPB, maximum plaque burden.

In 27 (40%) ruptured plaques, the distance from the proximal edge to the site of rupture was 23.5 ± 20.4 mm, to the maxNA site was 24.7 ± 20.8 mm, to the maxPB site was 28.3 ± 22.8 mm and to the MLA site was 34.0 ± 21.7 mm. The sites of rupture were closest to the maxNA sites (Fig 1B). The average distance from the site of rupture to the maxNA site was 0.33 ± 4.04 mm (Table 5). Simple linear regression analysis revealed close correlation between the sites of rupture and the maxNA sites (R^2^ = 0.94, *p* < 0.0001; Fig 1C). Specifically, 44% of ruptures were proximal to, 26% overlapped and 30% were distal to a maxNA site. In comparison with the closest correlation between the sites of rupture and maxNA sites, the correlation was weaker but significant between the sites of rupture and maxPB sites (R^2^ = 0.89, *p* < 0.0001; Fig 1D), and between the sites of rupture and MLA sites (R^2^ = 0.69, *p* < 0.0001; Fig 1E). Specifically, 70% of ruptures were proximal to, 11% overlapped and 19% were distal to a maxPB site, and 78% of ruptures were proximal to, 7% overlapped and 15% were distal to an MLA site (Table 5).

**Table 5:** Spatial relations between the sites of rupture, maxNA, maxPB and MLA sites.

Location of rupture	n (%)	Distance (mm)
To the maxNA site		
All	27 (100)	0.33 ± 4.04
Proximal to the maxNA site	12 (44)	3.00 ± 3.60
At the maxNA site	7 (26)	0
Distal to the maxNA site	8 (30)	3.38 ± 3.48
To the maxPB site		
All	27 (100)	3.32 ± 6.82
Proximal to the maxPB site	19 (70)	5.18 ± 7.37
At the maxPB site	3 (11)	0
Distal to of the maxPB site	5 (19)	1.73 ± 1.13
To the MLA site		
All	27 (100)	8.97 ± 11.40
Proximal to the MLA site	21 (78)	11.79 ± 11.45
At the MLA site	2 (7)	0
Distal to the MLA site	4 (15)	1.38 ± 0.46

Distances are mean ± SD for the ruptured lesions. maxNA, maximum necrotic area; maxPB, maximum plaque burden; MLA, minimum lumen area.

### Plaque characteristics in Ruptured Lesions and in TO Lesions

IVUS findings are shown for the ruptured and non-ruptured lesions in Table 6. Although the lumen volumes were comparable, the vessel and plaque volumes of ruptured lesions were significantly greater than those of non-ruptured lesions (vessel volume: 745.5 ± 41.3 mm^3^ versus 635.7 ± 33.5 mm^3^, *p* = 0.04; plaque volume: 450.0 ± 23.8 mm^3^ versus 387.7 ± 19.3 mm^3^, *p* = 0.046). With respect to plaque composition, the lipidic volume (57.6 ± 3.8 mm^3^ versus 46.9 ± 3.1 mm^3^, *p* = 0.03) of ruptured lesions was significantly greater than that of non-ruptured lesions, and the necrotic volume of the ruptured lesions tended to be greater (152.6 ± 12.1 mm^3^ versus 125.8 ± 9.8 mm^3^, *p* = 0.09).

**Table 6:** Intravascular ultrasound characteristics of the total lesions, ruptured and non-ruptured culprit lesions.

	All lesions	Ruptured lesions	Non-ruptured lesions	*p* Value*
	(n = 68)	(n = 27)	(n = 41)	
Length (mm)	48.75 ± 20.9	51.14 ± 4.03	47.18 ± 3.27	0.45
Thrombus, n (%)	52 (76)	18 (67)	32 (78)	0.3
Lesions with ≥2 ruptures, n	2 (3)	2 (7)	0	
Average plaque burden	0.61 ± 0.08	0.61 ± 0.01	0.61 ± 0.01	0.95
Maximum plaque burden	0.84 ± 0.06	0.85 ± 0.01	0.84 ± 0.01	0.31
Minimum lumen area (mm^2^)	2.21 ± 0.74	2.26 ± 0.14	2.17 ± 0.12	0.64
Lumen volume (mm^3^)	266.9 ± 124.8	295.5 ± 23.8	248.0 ± 19.3	0.13
EEM volume (mm^3^)	679.3 ± 219.7	745.5 ± 41.3	635.7 ± 33.5	**0.04**
Plaque volume (mm^3^)	412.4 ± 126.7	450.0 ± 23.8	387.7 ± 19.3	**0.046**
Composition volume (mm^3^)				
Fibrotic plaque	216.3 ± 67.5	230.4 ± 12.9	207.0 ± 10.5	0.16
Lipidic plaque	51.1 ± 20.4	57.6 ± 3.8	46.9 ± 3.1	**0.03**
Calcified plaque	6.6 ± 5.2	7.4 ± 1.0	6.1 ± 0.8	0.3
Necrotic plaque	126.5 ± 63.7	152.6 ± 12.1	125.8 ± 9.8	0.09

Values are mean ± SD unless otherwise indicated. Volumes were adjusted for lesion length. EEM, external elastic membrane.

*Ruptured versus non-ruptured.

The IVUS findings are shown for the TO lesions and non-TO lesions in Table 7. Lesion length was greater (54.16 ± 21.16 mm versus 40.01 ± 17.51 mm, *p* = 0.006), and the average PB and maxPB were greater (avgPB: 0.63 ± 0.07 versus 0.58 ± 0.08, *p* = 0.02; maxPB: 0.85 ± 0.06 versus 0.82 ± 0.06, *p* = 0.03) in the TO group than in the non-TO group. Thrombus was more prevalent in the TO group (83% versus 65%, *p* = 0.09). There was no significant between-group difference in vessel volume, lumen volume or plaque composition, and there was no significant difference in the prevalence of plaque rupture between the TO and non-TO lesions.

**Table 7:** Intravascular ultrasound features of TO lesions and non-TO lesions.

	TO lesions	Non-TO lesions	*p* Value
	(n = 42)	(n = 26)	
Length (mm)	54.16 ± 21.16	40.01 ± 17.51	**0.006**
Thrombus, n (%)	35 (83)	17 (65)	0.09
Plaque rupture, n (%)	17 (40)	10 (38)	0.87
Average plaque burden	0.63 ± 0.07	0.58 ± 0.08	**0.02**
Maximum plaque burden	0.85 ± 0.06	0.82 ± 0.06	**0.03**
Minimum lumen area (mm^2^)	1.99 ± 0.11	2.56 ± 0.14	**0.001**
Lumen volume (mm^3^)	250.8 ± 19.1	292.9 ± 24.3	0.18
EEM volume (mm^3^)	669.1 ± 34.1	695.8 ± 43.3	0.63
Plaque volume (mm^3^)	418.3 ± 19.7	403.0 ± 25.0	0.63
Composition volume (mm^3^)			
Fibrotic plaque	224.9 ± 10.4	202.3 ± 13.2	0.18
Lipidic plaque	50.2 ± 3.2	52.6 ± 4.0	0.65
Calcified plaque	6.1 ± 0.8	7.4 ± 1.0	0.31
Necrotic plaque	135.5 ± 10.0	138.1 ± 12.6	0.87

Values are mean ± SD unless otherwise indicated. Volumes were adjusted for lesion length. TO, totally occlusive; EEM, external elastic membrane.

## Discussion

Although maxNA, maxPB and MLA sites in AMI culprit lesions were shown to promote plaque rupture or blockage of blood flow and were recognized vulnerability, whether these sites overlap and the spatial correlations between the sites are still not known. This study investigated the spatial distribution of these sites and found these sites did not overlap in most cases. The sites of maxNA, maxPB and MLA originated proximally to distally within the lesions. The plaque ruptures originated mainly in the vicinity of the maxNA sites and proximal to the maxPB and MLA sites. This study also investigated the differences in plaque morphology and composition between TO and non-TO culprit lesions, and between ruptured and non-ruptured lesions in AMI patients. The TO culprit lesions, in comparison to the non-TO lesions, were longer and had a greater PB, but there was no difference in plaque composition. The ruptured lesions, in comparison to the non-ruptured lesions, were related to significantly greater vessel and plaque volumes and had significantly greater lipidic volume and somewhat greater necrotic volume.

In clinical practice, before the advent of IVUS, interventional cardiologists relied on angiographic identification of the narrowest part of a vessel as the focus for intervention therapy [25]. However, in the present study, we often found that the sites of greatest stenosis were not the sites of greatest danger. In most cases, the site of rupture was closest to the maxNA and proximal to narrowest part of the vessel. Although the results of a few previous studies indicated that the maxNA was often proximal to the most severe narrowing [26–28], we investigated the spatial distribution of the sites of rupture and found that, instead of the sites of maxPB or greatest narrowing, the sites of maxNA were at the greatest risk of plaque rupture. Although the locations of the sites of rupture correlated significantly with the locations of maxPB and greatest narrowing, most ruptures occurred proximal to the maxPB and MLA sites. Shear stress and fragility of the plaque surface may play important roles in plaque rupture. Increased shear stress has been found at the proximal part of the plaque hill [29,30], and when the shear stress meets with the fragile plaque surface, plaque rupture is initiated [31,32]. Thus, we hypothesize that the sites of maxNA, which are proximal to maxPB sites and MLA sites, may bear the highest stress and be the most fragile.

Interestingly, although the sites of rupture were closest to the maxNA sites, only 26% of ruptures occurred at the maxNA sites. The other 74% of ruptures occurred proximal (44%) or distal (30%) to the maxNA sites. These data indicate that the longitudinal “shoulder” of the maxNA might be the weakest spot. Prior studies of cross-sections have shown that the shoulder region of a plaque with a necrotic core is the most susceptible to rupture [33–35]. The current study extended this 2-dimensional concept to a 3-dimensional concept.

Plaque rupture with superimposed thrombosis plays a crucial role in the pathogenesis of AMI [36]. The frequencies of plaque rupture of culprit lesions in our AMI patients were 40%. The ruptured plaques and non-ruptured plaques had comparable lumen volumes but the vessel volumes of ruptured plaques were significantly increased. These results indicated that positive remodeling is a risk factor for plaque rupture [8,37–39]. Moreover, the plaques with greater lipidic and necrotic components were prone to rupture. Although it is widely acknowledged, on the basis of pathologic assessment at autopsy, that a large lipid pool or necrotic core covered by a thin fibrous cap is a feature of unstable plaque [40–42], *in vivo* studies investigating the composition of ruptured plaques are scarce. Our *in vivo* color-mapping IVUS imaging findings confirmed the pathologic findings and highlighted the significance of the various plaque components in plaque rupture. AMI patients with TO lesions, in comparison to AMI patients with non-TO lesions, have been shown to have larger infarcts and increased mortality as well as higher incidences of stent thrombosis and reinfarction [12–17]. Delayed revascularization, a lower PCI success rate and more severe clinical complications are assumed to be responsible for the poorer clinical outcomes in patients with TO lesions [12–14]. The present study provided some explanations for the serious clinical outcomes in patients with TO lesions. The longer lesions and greater PB suggest the development of more extensive and severe atherosclerotic plaques in the TO lesions [43]. Indeed, as in previously reported patient series [44,45], our patients with TO lesions often presented with STEMI, whereas our patients with non-TO lesions often presented with non-STEMI.

The present study was a single-center, retrospective observational study with a relative small population, which may introduce the selection bias. Further large prospective randomized studies are needed to validate our results. In addition to plaque rupture, plaque erosion is considered a major substrate for coronary thrombosis in AMI [46]. However, plaque erosion is difficult to identify by means of IVUS, which is inferior to intravascular optical coherence tomography (OCT). The characteristics of eroded plaques in AMI remain unclear. IVUS is also limited in its ability to identify all plaque ruptures and all thromboses [47]. Despite our best efforts, some microruptures without a typical residual fibrous cap and some fresh white microthrombi might have been overlooked. Thus, future studies incorporating both IVUS and OCT might be necessary. In the present study, 28% of the culprit lesions were predilated with a 1.5–2.0-mm balloon, and this could have led to overestimation of the vessel and lumen areas and underestimation of the plaque volume. Finally, plaque rupture is a complicated process involving both mechanical and biochemical factors [33,36,42,48]. Despite our understanding of the characteristics of ruptured plaques, it remains difficult to retrospectively trace the cascade of events and capture the core mechanisms underlying plaque rupture. Substantial cause-and-effect data derived from animal models that would elucidate the mechanisms of plaque rupture might be necessary.

## Conclusions

Instead of overlapping each other, most maxNA sites are proximal to the maxPB sites and MLA sites. The plaque ruptures originated mainly in the vicinity of the maxNA sites. Plaque morphology and composition play critical roles in plaque rupture and coronary occlusion.

## References

[1] Virmani R, Burke AP, Farb A, Kolodgie FD. Pathology of the vulnerable plaque. J Am Coll Cardiol. 2006;47:C13–C18. 16631505 10.1016/j.jacc.2005.10.065

[2] Davies MJ. The pathophysiology of acute coronary syndromes. Heart. 2000;83:361–366. 10677422 10.1136/heart.83.3.361 PMC1729334

[3] Toutouzas K, Karanasos A, Riga M, Tsiamis E, Synetos A, Michelongona A, et al Optical coherence tomography assessment of the spatial distribution of culprit ruptured plaques and thin-cap fibroatheromas in acute coronary syndrome. Eurointervention. 2012;8:477–485. 10.4244/EIJV8I4A75 22917732

[4] Kim SW, Hong YJ, Mintz GS, Lee SY, Doh JH, Lim SH, et al Relation of ruptured plaque culprit lesion phenotype and outcomes in patients with ST elevation acute myocardial infarction. Am J Cardiol. 2012;109:794–799. 10.1016/j.amjcard.2011.10.042 22196783

[5] Wykrzykowska JJ, Mintz GS, Garcia-Garcia HM, Maehara A, Fahy M, Xu K, et al Longitudinal distribution of plaque burden and necrotic core-rich plaques in nonculprit lesions of patients presenting with acute coronary syndromes. JACC Cardiovasc Imaging. 2012;5:S10–S18. 10.1016/j.jcmg.2012.01.006 22421223

[6] Finn AV, Nakano M, Narula J, Kolodgie FD, Virmani R. Concept of vulnerable/unstable plaque. Arterioscler Thromb Vasc Biol. 2010;30:1282–1292. 10.1161/ATVBAHA.108.179739 20554950

[7] Kusama I, Hibi K, Kosuge M, Sumita S, Tsukahara K, Okuda J, et al Intravascular ultrasound assessment of the association between spatial orientation of ruptured coronary plaques and remodeling morphology of culprit plaques in ST-elevation acute myocardial infarction. Heart Vessels. 2012;27:541–547. 10.1007/s00380-011-0184-7 21892739

[8] Ouldzein H, Elbaz M, Roncalli J, Cagnac R, Carrié D, Puel J, et al Plaque rupture and morphological characteristics of the culprit lesion in acute coronary syndromes without significant angiographic lesion: Analysis by intravascular ultrasound. Ann Cardiol Angeiol. 2012;61:20–26. 10.1016/j.ancard.2011.07.011 21903196

[9] Tanaka A, Shimada K, Yoshida K, Jissyo S, Tanaka H, Sakamoto M, et al Non-invasive assessment of plaque rupture by 64-slice multidetector computed tomography: comparison with intravascular ultrasound. Cir J. 2008;72:1276–1281. 10.1253/circj.72.1276 18654013

[10] Tian JW, Ren XF, Vergallo R, Xing L, Yu H, Jia H, et al Distinct morphological features of ruptured culprit plaque for acute coronary events compared to those with silent rupture and thin-cap fibroatheroma a combined optical coherence tomography and intravascular ultrasound study. J Am Coll Cardiol. 2014;63:2209–2216. 24632266 10.1016/j.jacc.2014.01.061

[11] von Birgelen C, Klinkhart W, Mintz GS, Papatheodorou A, Herrmann J, Baumgart D, et al Plaque distribution and vascular remodeling of ruptured and nonruptured coronary plaques in the same vessel: an intravascular ultrasound study in vivo. J Am Coll Cardiol. 2001;37:1864–1870. 11401124 10.1016/s0735-1097(01)01234-7

[12] Wang TY, Zhang M, Fu YL, Armstrong PW, Newby LK, Gibson CM, et al Incidence, distribution, and prognostic impact of occluded culprit arteries among patients with non-ST-elevation acute coronary syndromes undergoing diagnostic angiography. Am Heart J. 2009;157:716–723. 10.1016/j.ahj.2009.01.004 19332201

[13] Bahrmann P, Rach J, Desch S, Schuler GC, Thiele H. Incidence and distribution of occluded culprit arteries and impact of coronary collaterals on outcome in patients with non-ST-segment elevation myocardial infarction and early invasive treatment strategy. Clin Res Cardiol. 2011;100:457–467. 10.1007/s00392-010-0269-9 21165625

[14] Kim MC, Ahn Y, Rhew SH, Jeong MH, Kim JH, Hong YJ, et al; KAMIR Investigators. Impact of total occlusion of an infarct-related artery on long-term mortality in acute non-ST-elevation myocardial infarction patients who underwent early percutaneous coronary intervention. Int Heart J. 2012;53:160–164. 22790683 10.1536/ihj.53.160

[15] Piccolo R, Galasso G, Iversen AZ, Eitel I, Dominguez-Rodriguez A, Gu YL, et al Effects of baseline coronary occlusion and diabetes mellitus in patients with ST-segment elevation myocardial infarction undergoing primary percutaneous coronary intervention. Am J Cardiol. 2014;114:1145–1150. 10.1016/j.amjcard.2014.07.030 25193670

[16] Shin DI, Chang K, Ahn Y, Hwang BH, Park HJ, Seo SM, et al Impact of occluded culprit arteries on long-term clinical outcome in patients with non-ST-elevation myocardial infarction: 48-month follow-up results in the COREA-AMI Registry. J Interv Cardiol. 2014;27:12–20. 10.1111/joic.12078 24147831

[17] Pride YB, Tung P, Mohanavelu S, Zorkun C, Wiviott SD, Antman EM, et al; TIMI Study Group. Angiographic and clinical outcomes among patients with acute coronary syndromes presenting with isolated anterior ST-segment depression a TRITON TIMI 38 (trial to assess improvement in therapeutic outcomes by optimizing platelet inhibition with prasugrel thrombolysis in myocardial infarction 38) substudy. JACC Cardiovasc Interv. 2010;3:806–811. 20723851 10.1016/j.jcin.2010.05.012

[18] Shin ES, Garcia-Garcia HM, Ligthart JM, Witberg K, Schultz C, van der Steen AF, et al In vivo findings of tissue characteristics using iMAP (TM) IVUS and Virtual Histology (TM) IVUS. Eurointervention. 2011;6:1017–1019. 10.4244/EIJV6I8A175 21330252

[19] Heo JH, Brugaletta S, Garcia-Garcia HM, Gomez-Lara J, Ligthart JM, Witberg K, et al Reproducibility of intravascular ultrasound iMAP for radiofrequency data analysis: implications for design of longitudinal studies. Catheter Cardiovasc Interv. 2014;83:E233–E242. 10.1002/ccd.23335 22109902

[20] Yamada R, Okura H, Kume T, Neishi Y, Kawamoto T, Miyamoto Y, et al A comparison between 40 MHz intravascular ultrasound iMap imaging system and integrated backscatter intravascular ultrasound. J Cardiol. 2013;61:149–154. 10.1016/j.jjcc.2012.10.008 23265675

[21] Mintz GS, Nissen SE, Anderson WD, Bailey SR, Erbel R, Fitzgerald PJ, et al American College of Cardiology clinical expert consensus document on standards for acquisition, measurement and reporting of intravascular ultrasound studies (IVUS). A report of the American College of Cardiology Task Force on Clinical Expert Consensus Documents. J Am Coll Cardiol. 2001;37:1478–1492. 11300468 10.1016/s0735-1097(01)01175-5

[22] Xie Y, Mintz GS, Yang JQ, Doi H, Iñiguez A, Dangas GD, et al Clinical outcome of nonculprit plaque ruptures in patients with acute coronary syndrome in the PROSPECT study. JACC Cardiovasc Imaging. 2014;7:397–405. 10.1016/j.jcmg.2013.10.010 24631511

[23] Garcia-Garcia HM, Gogas BD, Serruys PW, Bruining N. IVUS-based imaging modalities for tissue characterization: similarities and differences. Int J Cardiovasc Imaging. 2011;27:215–224. 10.1007/s10554-010-9789-7 21327914 PMC3078312

[24] Nissen SE, Tuzcu EM, Schoenhagen P, Crowe T, Sasiela WJ, Tsai J, et al; Reversal of Atherosclerosis with Aggressive Lipid Lowering (REVERSAL) Investigators. Statin therapy, LDL cholesterol, C-reactive protein, and coronary artery disease. N Engl J Med. 2005;352:29–38. 15635110 10.1056/NEJMoa042000

[25] Kushner FG, Hand M, Smith SC Jr, King SB 3rd, Anderson JL, Antman EM, et al 2009 focused updates: ACC/AHA guidelines for the management of patients with ST-elevation myocardial infarction (updating the 2004 guideline and 2007 focused update) and ACC/AHA/SCAI guidelines on percutaneous coronary intervention (updating the 2005 guideline and 2007 focused update) a report of the American College of Cardiology Foundation/American Heart Association Task Force on Practice Guidelines. J Am Coll Cardiol. 2009;54:2205–2241. 19942100 10.1016/j.jacc.2009.10.015

[26] de Graaf MA, van Velzen JE, de Graaf FR, Schuijf JD, Dijkstra J, Bax JJ, et al The maximum necrotic core area is most often located proximally to the site of most severe narrowing: a virtual histology intravascular ultrasound study. Heart Vessels. 2013;28:166–172. 10.1007/s00380-012-0236-7 22349692

[27] Kaple RK, Maehara A, Sano K, Missel E, Castellanos C, Tsujita K, et al The axial distribution of lesion-site atherosclerotic plaque components: an in vivo volumetric intravascular ultrasound radio-frequency analysis of lumen stenosis, necrotic core and vessel remodeling. Ultrasound Med Biol. 2009;35:550–557. 10.1016/j.ultrasmedbio.2008.09.024 19110364

[28] Legutko J, Jakala J, Mintz GS, Kaluza GL, Mrevlje B, Partyka L, et al Radiofrequency-intravascular ultrasound assessment of lesion coverage after angiography-guided emergent percutaneous coronary intervention in patients with non-ST elevation myocardial infarction. Am J Cardiol. 2013;112:1854–1859. 10.1016/j.amjcard.2013.08.011 24063826

[29] Li ZY, Gillard JH. Plaque rupture: plaque stress, shear stress, and pressure drop. J Am Coll Cardiol. 2008;52:499–500. 10.1016/j.jacc.2008.04.040 18672176

[30] Li ZY, Howarth SPS, Tang T, Gillard JH. How critical is fibrous cap thickness to carotid plaque stability? A flow-plaque interaction model. Stroke. 2006;37:1195–1199. 16574926 10.1161/01.STR.0000217331.61083.3b

[31] Speelman L, Akyildiz AC, den Adel B, Wentzel JJ, van der Steen AF, Virmani R, et al Initial stress in biomechanical models of atherosclerotic plaques. J Biomech. 2011;44:2376–2382. 10.1016/j.jbiomech.2011.07.004 21782179

[32] Tang DL, Teng ZZ, Canton G, Yang C, Ferguson M, Huang X, et al Sites of rupture in human atherosclerotic carotid plaques are associated with high structural stresses an in vivo MRI-based 3D fluid-structure interaction study. Stroke. 2009;40:3258–3263. 10.1161/STROKEAHA.109.558676 19628799 PMC2753753

[33] Fishbein MC. The vulnerable and unstable atherosclerotic plaque. Cardiovasc Pathol. 2010;19:6–11. 10.1016/j.carpath.2008.08.004 18835793

[34] Toutouzas K, Karanasos A, Tsiamis E, Riga M, Drakopoulou M, Synetos A, et al New insights by optical coherence tomography into the differences and similarities of culprit ruptured plaque morphology in non-ST-elevation myocardial infarction and ST-elevation myocardial infarction. Am Heart J. 2011;161:1192–1199. 10.1016/j.ahj.2011.03.005 21641368

[35] Plutzky J. Atherosclerotic plaque rupture: Emerging insights and opportunities. Am J Cardiol. 1999;84:15J–20J. 10418853 10.1016/s0002-9149(99)00352-5

[36] Falk E, Nakano M, Bentzon JF, Finn AV, Virmani R. Update on acute coronary syndromes: the pathologists’ view. Eur Heart J. 2013;34:719–728. 10.1093/eurheartj/ehs411 23242196

[37] Kubo T, Imanishi T, Takarada S, Kuroi A, Ueno S, Yamano T, et al Assessment of culprit lesion morphology in acute myocardial infarction: ability of optical coherence tomography compared with intravascular ultrasound and coronary angioscopy. J Am Coll Cardiol. 2007;50:933–939. 17765119 10.1016/j.jacc.2007.04.082

[38] Topaz O, Bernardo NL, Shah R, McQueen RH, Desai P, Janin Y, et al Effectiveness of excimer laser coronary angioplasty in acute myocardial infarction or in unstable angina pectoris. Am J Cardiol. 2001;87:849–855. 11274939 10.1016/s0002-9149(00)01525-3

[39] Takumi T, Lee S, Hamasaki S, Toyonaga K, Kanda D, Kusumoto K, et al Limitation of angiography to identify the culprit plaque in acute myocardial infarction with coronary total occlusion utility of coronary plaque temperature measurement to identify the culprit plaque. J Am Coll Cardiol. 2007;50:2197–2203. 18061065 10.1016/j.jacc.2007.07.079

[40] Uemura S, Ishigami K, Soeda T, Okayama S, Sung JH, Nakagawa H, et al Thin-cap fibroatheroma and microchannel findings in optical coherence tomography correlate with subsequent progression of coronary atheromatous plaques. Eur Heart J. 2012;33:78–85. 10.1093/eurheartj/ehr284 21831910

[41] Virmani R, Kolodgie FD, Burke AP, Finn AV, Gold HK, Tulenko TN, et al Atherosclerotic plaque progression and vulnerability to rupture: angiogenesis as a source of intraplaque hemorrhage. Arterioscler Thromb Vasc Biol. 2005;25:2054–2061. 16037567 10.1161/01.ATV.0000178991.71605.18

[42] Bentzon JF, Otsuka F, Virmani R, Falk E. Mechanisms of plaque formation and rupture. Circ Res. 2014;114:1852–1866. 10.1161/CIRCRESAHA.114.302721 24902970

[43] Nicholls SJ, Hsu A, Wolski K, Hu B, Bayturan O, Lavoie A, et al Intravascular ultrasound-derived measures of coronary atherosclerotic plaque burden and clinical outcome. J Am Coll Cardiol. 2010;55:2399–2407. 10.1016/j.jacc.2010.02.026 20488313

[44] Chan MY, Sun JL, Newby LK, Shaw LK, Lin M, Peterson ED, et al Long-term mortality of patients undergoing cardiac catheterization for ST-elevation and non-ST-elevation myocardial infarction. Circulation. 2009;119:3110–3117. 10.1161/CIRCULATIONAHA.108.799981 19506116

[45] van Leeuwen MAH, Daemen J, van Mieghem NM, de Boer SP, Boersma E, van Geuns RJ, et al; Interventional Cardiologists of the Thoraxcenter 2000–2009. Comparison of long-term outcomes in STEMI and NSTE-ACS after coronary stent placement: an analysis in a real world BMS and DES population. Int J Cardiol. 2013;167:2082–2087. 10.1016/j.ijcard.2012.05.064 22664371

[46] Arbustini E, Dal Bello B, Morbini P, Burke AP, Bocciarelli M, Specchia G, et al Plaque erosion is a major substrate for coronary thrombosis in acute myocardial infarction. Heart. 1999;82:269–272. 10455073 10.1136/hrt.82.3.269 PMC1729173

[47] Nissen SE, Yock P. Intravascular ultrasound: novel pathophysiological insights and current clinical applications. Circulation. 2001;103:604–616. 11157729 10.1161/01.cir.103.4.604

[48] Shah PK. Mechanisms of plaque vulnerability and rupture. J Am Coll Cardiol. 2003;41:15S–22S. 12644336 10.1016/s0735-1097(02)02834-6

